# Bibliometric analysis of fourth industrial revolution applied to heritage studies based on web of science and scopus databases from 2016 to 2021

**DOI:** 10.1186/s40494-022-00821-3

**Published:** 2022-11-22

**Authors:** Anibal Alviz-Meza, Manuel H. Vásquez-Coronado, Jorge G. Delgado-Caramutti, Daniel J. Blanco-Victorio

**Affiliations:** 1 Grupo de Investigación en Deterioro de Materiales, Transición Energética y Ciencia de Datos DANT3, Faculty of Enginering, Arquitecture and Urbanism, Universidad Señor de Sipán, Chiclayo, Pimentel 14001 Peru; 2 Chemical Engineering School, Universidad de Cartagena, Avenida del Consulado #Calle, 30 No. 48 152, Cartagena de Indias, Colombia; 3 Semillero de Investigación en Corrosión de Metales, Energías Sostenibles y Análisis de Datos - COMD3S, Faculty of Enginering, Arquitecture and Urbanism, Universidad Señor de Sipán, Chiclayo, Pimentel 14001 Peru; 4 INVESSALUD, Life sciences and human health care, Universidad Señor de Sipán, Chiclayo, Pimentel 14001 Peru

**Keywords:** Bibliometric, Heritage, Industry 4.0, Scopus, Web of Science, Biblioshiny, VOSviewer

## Abstract

Using past material and spiritual remains, cultural heritage examines communities’ identity formation across time. Cultural heritage requires public and private institutions to care about its restoration, maintenance, conservation, and promotion. Through a bibliometric perspective, this study has analyzed, quantified, and mapped the scientific production of the fourth industrial revolution applied to heritage studies from 2016 to 2021 in the Scopus and Web of Science databases. Biblioshiny software from RStudio was employed to categorize and evaluate the contribution of authors, countries, institutions, and journals. In addition, VOSviewer was used to visualize their collaboration networks. As a main result, we found that augmented reality and remote sensing represent the research hotspot concerning heritage studies. Those techniques have become common in archaeology, as well as museums, leading to an increase in their activity. Perhaps, more recent tools, such as machine learning and deep learning, will provide future pathways in cultural heritage from data collected in social networks. This bibliometric analysis, therefore, provides an updated perspective of the implementations of technologies from industry 4.0 in heritage science as a possible guideline for future worldwide research.

## Introduction

Cultural heritage studies have become relevant worldwide due to their impact on the revaluation of human history, leading to initiatives to increase social and economic impacts in several countries. In recent years, heritage studies have started to be boosted by technologies from the fourth industrial revolution -industry 4.0- such as the internet of things (IoT), artificial intelligence, augmented reality, remote sensing, etc., extending the known horizons of the field.

These technologies have varied non-invasive applications for heritage sites, as the following examples demonstrate. IoT has enabled the conservation of cultural heritage by managing data collected by sensors and by enhancing the interaction and experience of visitors with cultural objects as much as by preserving the related records [1–3]. Additionally, three-dimensional documentation of cultural heritage has been used to fully visualize tall and heavy sculptures through aerial laser scanners and photographs, without which it would not have been possible to be observed [4]. Likewise, artistic heritage can be digitalized with slow-motion technology techniques [5].

Another emerging technology in cultural tourism is augmented reality (AR), which improves consumer satisfaction with the introduction of easy-to-use technological devices, rich in information easier to catch for the public [6]. Moreover, most tourists have claimed that meaningful designs, valuable contents, and intuitive interfaces are key factors for successful AR mobile apps [7]. The use of machine learning techniques has made it feasible for the preservation of intangible heritage such as cultural dances; in such cases, dancers’ movements have been captured with graphics to visually preserve, analyze and understand choreographic patterns [8]. Meanwhile, satellite remote sensing has allowed the discovery of historical places as well as the creation of strategies for their conservation and management by identifying factors that may endanger them in the future [9, 10].

On the other hand, bibliometric analysis has been increasing in heritage studies associated with computer sciences, focusing on industrial heritage [11], intangible heritage [12], and creative and cultural heritages [13–15]. Bibliometrics has primarily been used to identify industry 4.0 as a disruptive phenomenon with potential implications in various disciplines and production processes [16]. Limited attention has been paid to bibliometric analysis based on the recent timeframe 2016–2021. Using the software biblioshiny we were able to collect a large number of articles, from Scopus and Web of Science (WoS) databases, across various emerging topics for analysis. Therefore, this research aims to compile and process-wide data associated with technologies from industry 4.0 applicated to heritage studies in the last six years. The following research questions were addressed:

Q1: How many research articles were annually published between 2016 and 2021 in industry 4.0 that were applied to heritage science?

Q2: Who are the most cited authors in studies associated with industry 4.0?

Q3: Which papers are the most cited in heritage studies in conjunction with industry 4.0?

Q4: Which journals host the highest quantities of papers in this research area?

Q5: What leaders’ institutions are found in the focused research field?

Q6: What sponsor institutions are the most active in the selected period?

Q7: What are the top ten countries publishing on this subject?

Bibliometric research can lead to the development and discovery of trends in a field, helping the scientific community to identify new hotbeds of innovation based on a recent window of observation [17]. This bibliometric analysis provides an updated perspective of the implementations of technologies from industry 4.0 in heritage research as a scientific reference for subsequent research. We provide the latest progress related to research, hotspots, and future possible development trends.

## Methodology

### Study design

This study employed a bibliometric analysis as a tool commonly used to map research in indistinct academic fields. This area is also named scientometrics, which uses math and statistic to numerically describe the scientific activity and relevance across a certain period [17].

### Data source

Scopus and Web of Science databases were selected due to their wide renown for hosting high-quality journals and research documents. Institutional access was required to download and corroborate the content of the study files.

### Search strategy

**Fig. 1:**
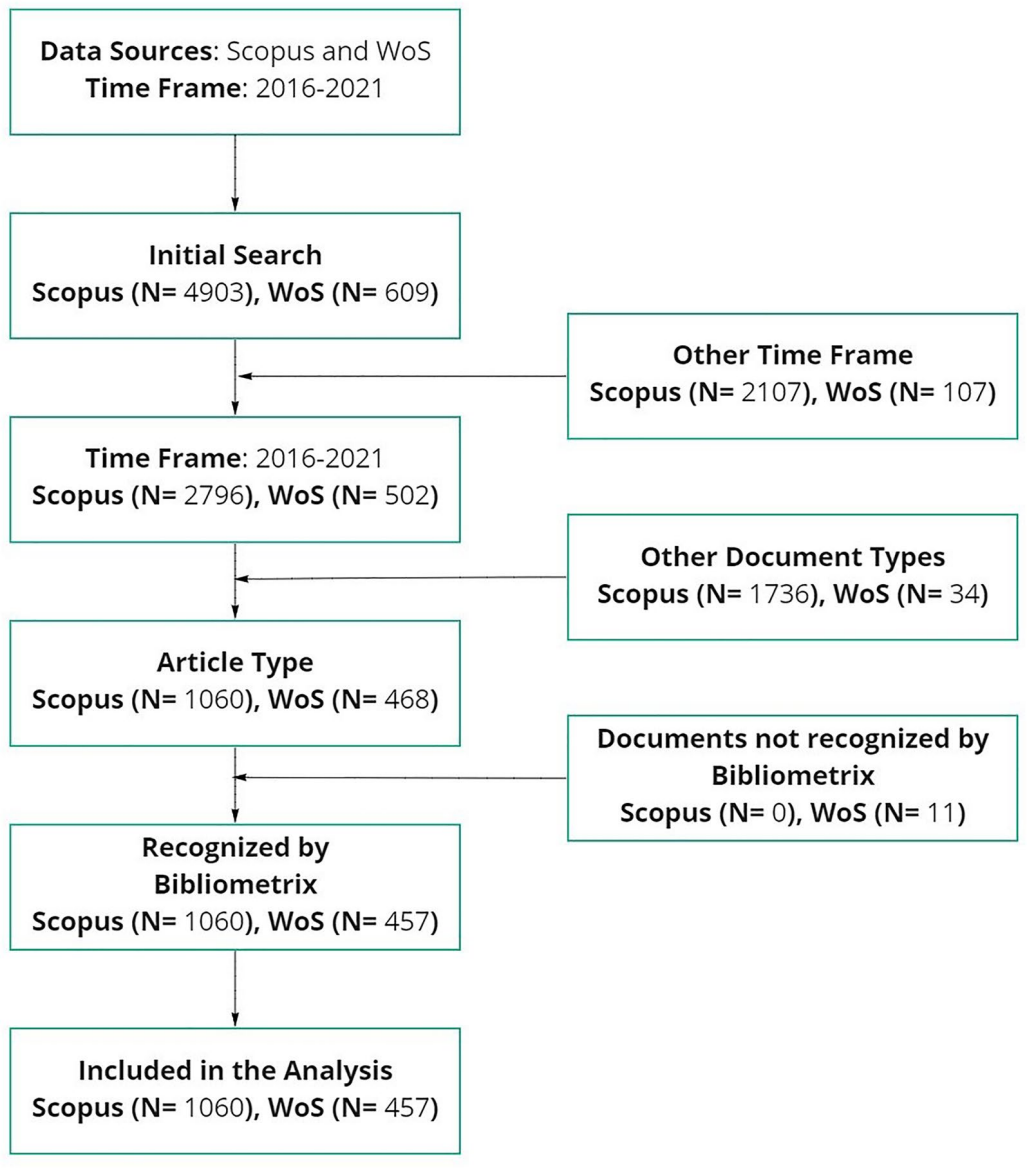
Flowchart of used bibliometric methodology

We introduced an extended list of keywords in both databases, covering heritage and industry 4.0 topics (see Fig. 1). On behalf of industry 4.0, the words used were the following: data science, industry 4.0, augmented reality, computer science, remote sensing, artificial intelligence, 3D scanning, data mining, data analytics, data handling, data processing, big data, data visualization, internet of things, and machine learning. Representing heritage areas, the selected words were the following: heritage, museums, monuments, paintings, conservatory, archeology, cultural tourism, art restoration, arts and humanities, preventive conservation, collections care, and art conservation. These keywords were obtained in a cyclical process in which, starting from the articles thrown by the databases, more words were incorporated, covering initially unforeseen topics. The established timeline covered data between 2016 and 2021 while the search was reduced to titles and keywords to increase the effectiveness of the equation to collect papers from the objective fields. Only original articles were considered as the document type. Both web pages were consulted for the last time on June 15th, 2022.

### Bibliometric analysis

Plots and tables combined the separately processed and analyzed data downloaded in *BibTeX* files from Scopus and WoS databases. The *Biblioshiny* app from the *RStudio* cloud served as a tool to obtain and organize both databases prior to manual manipulation. *Biblioshiny* offers data about the most productive countries, institutions, authors, research fields, and journals, as well as about keywords, h-index, impact factor, total citations, etc. [18]. Moreover, VOSviewer was included for data mining, mapping, and visualization of collaborative networks [19].

### Limitations

Scopus and WoS databases are not perfectly adapted to bibliometric analyses, since they tend to throw up a certain amount of erroneous data that limits the conclusions to be drawn from them. Qualitative statements can be subjective since this type of study is quantitative [20]. This type of academic exercise offers a short-term forecast of the area under investigation [21].

## Results and discussion

The arguments about the established objectives are addressed in the upcoming subsections, based on the data taken from WoS and Scopus, which indicates that Scopus is the preferred database to spread research articles related to industry 4.0 in the heritage studies, largely doubling the number of documents hosted in WoS (see Fig. 1).

### Trends in the annual production of original papers

**Fig. 2:**
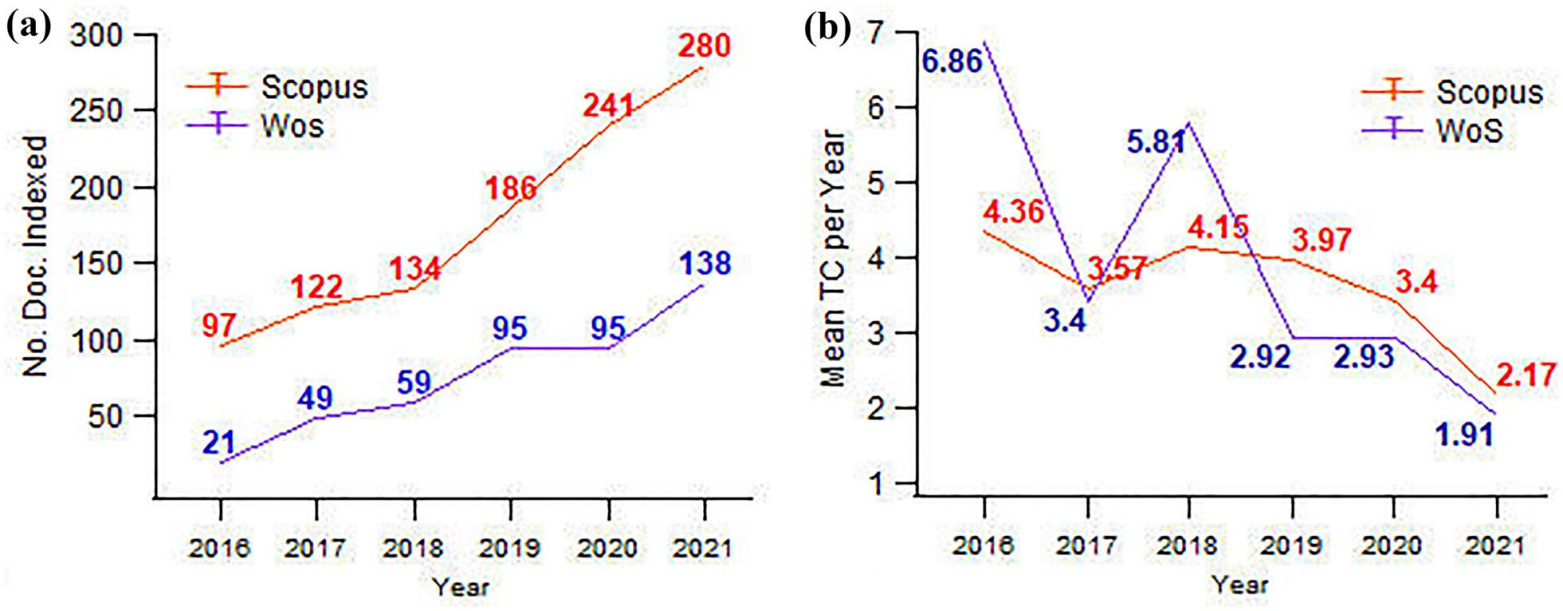
Annual trend of publication **a** and total citation **b** from WoS and Scopus from 2016 to 2021

Although the number of documents indexed in Scopus was found higher than in WoS in the studied timeframe, the overall average of total citations is slightly higher in WoS compared to Scopus, reaching 3.98 citations per year against the 3.60 of Scopus (see Fig. 2). In both cases, the research production represents a linear sustained growth -as expected with the apparition of new technologies in heritage science [22]- with a larger jump in productivity from 2019 to 2020 in Scopus, and from 2020 to 2021 in WoS. One of the likely reasons for these increments is related to the incorporation of new technologies into cultural tourism [23], and the opening of access to journals during the COVID-19 pandemic [24]. Meanwhile, the decay of total citations (TC) over years may respond to a phenomenon associated with the time required by researchers to identify the newly published works, their novelty, and their accessibility, in combination with today’s science productivity, etc. [25–27].

### Most cited authors and their collaborations

**Fig. 3:**
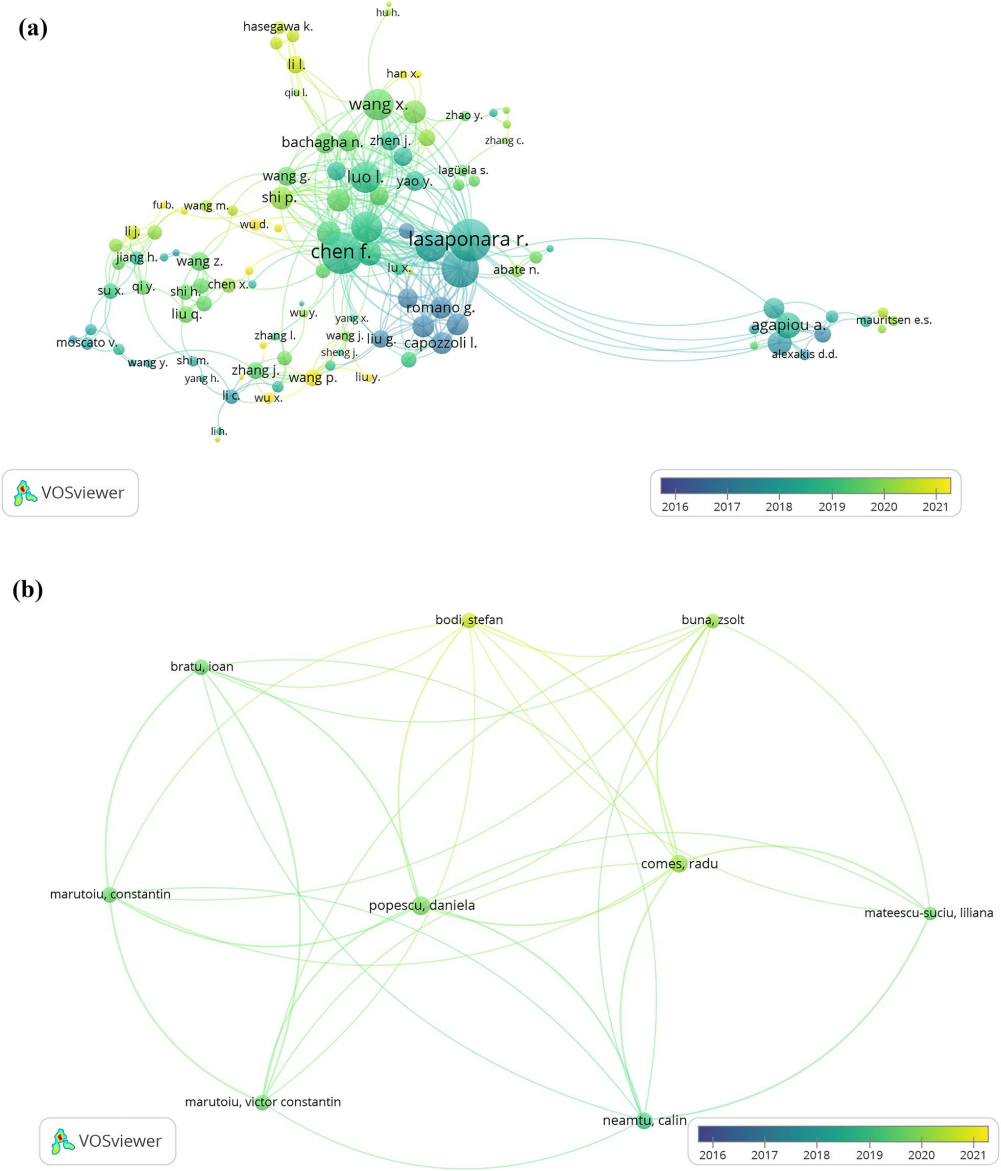
Most collaborative authors in heritage studies combined with industry 4.0 from 2016 to 2021 in **a** Scopus and **b** WoS, considering a minimum of two documents per institution in VOSviewer

Researchers’ performances in a given field are usually quantitatively measured through indicators such as the number of published papers, total citations, h-index, and other derivatives [28]. In this case, Scopus and WoS databases coincide in the conclusion that Piccialli F. is one of the more productive researchers in heritage studies combined with technologies from industry 4.0 (see Table 1). As a general observation of the data, Agapiou A. and Lasaponara R. have delivered the largest quantity of articles whereas Dieck MC. is the author with the most elevated number of citations. As shown in Fig. 3, Lasaponara R. is the researcher with the most collaborations (67) in Scopus, followed by Chen F. (63), and Massini N. (52). Piccialli F., however, stands out as the author with greatest link strength from WoS, with 16 connections (see Table 7 from appendices). These findings, including the h-index, may indicate that Lasaponara R. is the most influential author in studies developed around industry 4.0 and its application to heritage concerns. It is worth noting that images from Fig. 3 are not tailored to the data of Table 1 since these networks’ charts are focused on searching for collaborations -total link strength- and they depend on the minimum article per author selected and on the decision of presenting the interconnection of nodes. In this case, the node size is proportional to the number of authors’ partnerships. The same logic applies to the following VOSviewer figures and analysis along with the document.

**Table 1:** Top 10 most cited authors in heritage-industry 4.0 research (2016–2021)

Rank	Scopus				WoS			No. of paper
	Author	h-index	TC	No. of paper	Author	h-index	TC	
1st	Agapiou A	9	261	17	Dieck MC	8	587	8
2nd	Lasaponara R	12	447	16	Chianese A	5	111	7
3rd	Piccialli F	8	315	15	Piccialli F	5	74	6
4th	Chen F	7	280	13	Jung T	5	299	5
5th	Masini N	10	386	11	Lasaponara R	4	63	5
6th	Dieck MC	9	752	11	Wang M	3	20	5
7th	Chianese A	5	171	8	Wang X	2	10	5
8th	Hadjimitsis Dg	7	174	8	Agapiou A	4	89	4
9th	Wang X	4	136	8	Cejka J	3	35	4
10th	Cuomo S	4	137	7	Cigna F	4	149	4

### Most cited research articles

As shown in Table 2, the three most cited papers were written by Sun Y. [31], Dieck MC., and Alletos S. The number of citations observed in both databases is similar, highlighting the work of Sun Y. [31] with 476 average total citations, considering both databases. Augmented reality and the internet of things are subjects of great interest in these publications, with 6 and 2 published works from the top ten, respectively. The use of augmented reality in cultural heritage has grown notably in recent years due to its great public appeal [29]. People enjoy the interactive experience provided by digital tools, which translate the content of high scientific value into easily understandable elements, making it more attractive to society [30]. As previously mentioned, IoT contributes to the conservation of cultural heritage -sustainability- by assisting the management of data collected from sensors, as better explained in the work of Sun Y. [1, 31].

**Table 2:** Top 10 most cited articles in heritage-industry 4.0 research (2016–2021)

Autor, year	Document title and journal name	Journal name	TC Scopus	TC WoS
Sun Y, 2016	Internet of things and big data analytics for smart and connected communities	IEEE Access	536	415
Dieck MC, 2018	A theoretical model of mobile augmented reality acceptance in urban heritage tourism	Curr Issues Tour	174	170
Alletto S, 2016	An indoor location-aware system for an IoT-based smart museum	IEEE Internet Things J	174	138
He Z, 2018	When art meets tech: The role of augmented reality in enhancing museum experiences and purchase intentions	Tour Manage	110	89
Dieck MC, 2017	Value of augmented reality at cultural heritage sites: A stakeholder approach	J Destin Mark Manage	108	93
Jung TH, 2017	Augmented reality, virtual reality, and 3D printing for the co-creation of value for the visitor experience at cultural heritage places	J Place Manage Dev	106	89
Chung N, 2018	The role of augmented reality for experience-influenced environments: the case of cultural heritage tourism in Korea	J Travel Res	105	92
Luo L, 2019	Airborne and spaceborne remote sensing for archaeological and cultural heritage applications: a review of the century (1907–2017)	Remote Sens Environ	97	1
Ofli F, 2016	Combining human computing and machine learning to make sense of big (aerial) data for disaster response	Big Data	91	N/A
Han DI, 2018	User experience model for augmented reality applications in urban heritage tourism	J Herit Tour	85	80

### Journals that host the highest number of articles

According to both, Scopus and WoS databases, Table 3 shows that the journals with the most participation in heritage studies including tools from industry 4.0 are *Remote sensing* from the United States, *Sustainability* from Switzerland, and *Journal on computing and cultural heritage* from the United States. Approximately, 15% of the published articles in the studied fields are hosted in said three journals from both databases. That is, there exists a large spectrum of journals (85%) in which to publish articles regarding heritage issues mixed with computer science technologies. Moreover, most of the topics covered by the journals’ scope are related to the deterioration of attractions, monuments, artifacts, and places of great heritage value, aiming to contribute to their reconstruction and preservation [32, 33], as well as to encourage presential and virtual tourism [34, 35].

**Table 3:** Top 10 Journals that hosted the most articles in heritage-industry 4.0 research (2016–2021)

Rank	Scopus			WoS		
	Journal name	No. of papers (%) N = 1060	Impact factor SJR (2021)	Journal name	No. of papers (%) N = 468	Impact factor JCR (2021)
1st	Remote sensing	85 (8.02)	1.28	Sustainability (Switzerland)	25 (5.34)	3.89
2nd	Sustainability (Switzerland)	36 (3.4)	0.66	Remote sensing	24 (5.13)	5.35
3rd	Journal on computing and cultural heritage	35 (3.30)	0.81	Journal on computing and cultural heritage	21 (4.49)	2.05
4th	Applied sciences (Switzerland)	21 (1.98)	0.51	Applied sciences (Switzerland)	19 (4.06)	2.84
5th	Journal of archaeological science	19 (1.79)	1.44	IEEE Access	14 (2.99)	3.48
6th	Sensors (Switzerland)	18 (1.70)	0.80	Journal of cultural heritage	11 (2.35)	3.23
7th	Geosciences (Switzerland)	17 (1.60)	0.64	ISPRS International journal of geo-information	9 (1.92)	3.10
8th	IEEE Access	17 (1.60)	0.93	Journal of heritage tourism	9 (1.92)	N/A
9th	Journal of archaeological science: reports	15 (1.42)	0.73	Sensors (Switzerland)	9 (1.92)	3.85
10th	Journal of cultural heritage	15 (1.42)	0.72	Multimedia tools and applications	7 (1.50)	2.58

Through introducing Bradford's law, it was feasible to classify sources into core areas, related areas, and not relevant areas regarding the field addressed by the articles hosted in the journals, as expressed in Eq. (1). This law emerges as a plausible indicator to explain why many authors prefer to publish their research works in the journals presented in Table 3.

1 $${r}_{0}=2\mathrm{ln}({e}^{\gamma }Y)$$

where ${r}_{0}$ represents the number of journals conforming the core area, $\gamma$ is the Euler’s constant $(\sim 0.577)$, and $Y$ is the number of papers in the journal with more hosted documents [36]. In this case, since we are studying two databases, ${Y}_{1}=85$ in Scopus and ${Y}_{2}=25$ in WoS. Thus, ${r}_{0-1}\left(Scopus\right)\cong 10$ and ${r}_{0-2}\left(WoS\right)\cong 8$. As a result, only the source *Multimedia tools and applications from* WoS is out of the core collection, considering that *Sensors* is into the Scopus list. Also, it is notable that *Remote sensing* is the preferred journal to publish articles around industry 4.0 linked with heritage studies.

### Most productive institutions and their collaborations

Scopus and WoS provided different results regarding the most productive institutions, considering at least two publications per institute in VOSviewer while omitting unconnected nodes. The top 3 most productive universities in Scopus publish less than in the WoS case, positioning the University of Naples Federico II as the most contributive institution; followed by Cyprus and Manchester Universities (see Table 4). The success of the University of Naples Federico II in this field may be explained by initiatives such as the creation of the”High-Tech FabLab DIETI Unina” (HT FabLab) in recent years, which aimed to spread high-technology digital design/implementation to students through the development of practical projects for their training and research initiatives [37].

**Table 4:** Top 10 most productive institutions of heritage-industry 4.0 research (2016–2021)

Rank	Scopus			WoS		
	Affiliations	Country	No. of paper	Affiliations	Country	No. of paper
1st	Cyprus University of Technology	Cyprus	23	University of Naples Federico II	Italy	26
2nd	University of Naples Federico II	Italy	17	Manchester Metropolitan University	UK	20
3rd	University of Padova	Italy	17	Cyprus University of Technology	Cyprus	16
4th	University of Chinese Academy of Sciences	China	16	Universitat Politècnica De València	Spain	16
5th	Institute of Remote Sensing and Digital Earth	China	15	Università Politecnica Delle Marche	Italy	14
6th	Manchester Metropolitan University	UK	15	Delft University of Technology	Nether-lands	13
7th	University of Cambridge	UK	15	Lublin University of Technology	Poland	13
8th	Ghent University	Belgium	13	Kyung Hee University	S Korea	12
9th	Universitat Politècnica de València	Spain	12	Chung-Ang University	S Korea	11
10th	University of Salamanca	Spain	12	Technical University of Cluj-Napoca	Romania	10

**Fig. 4:**
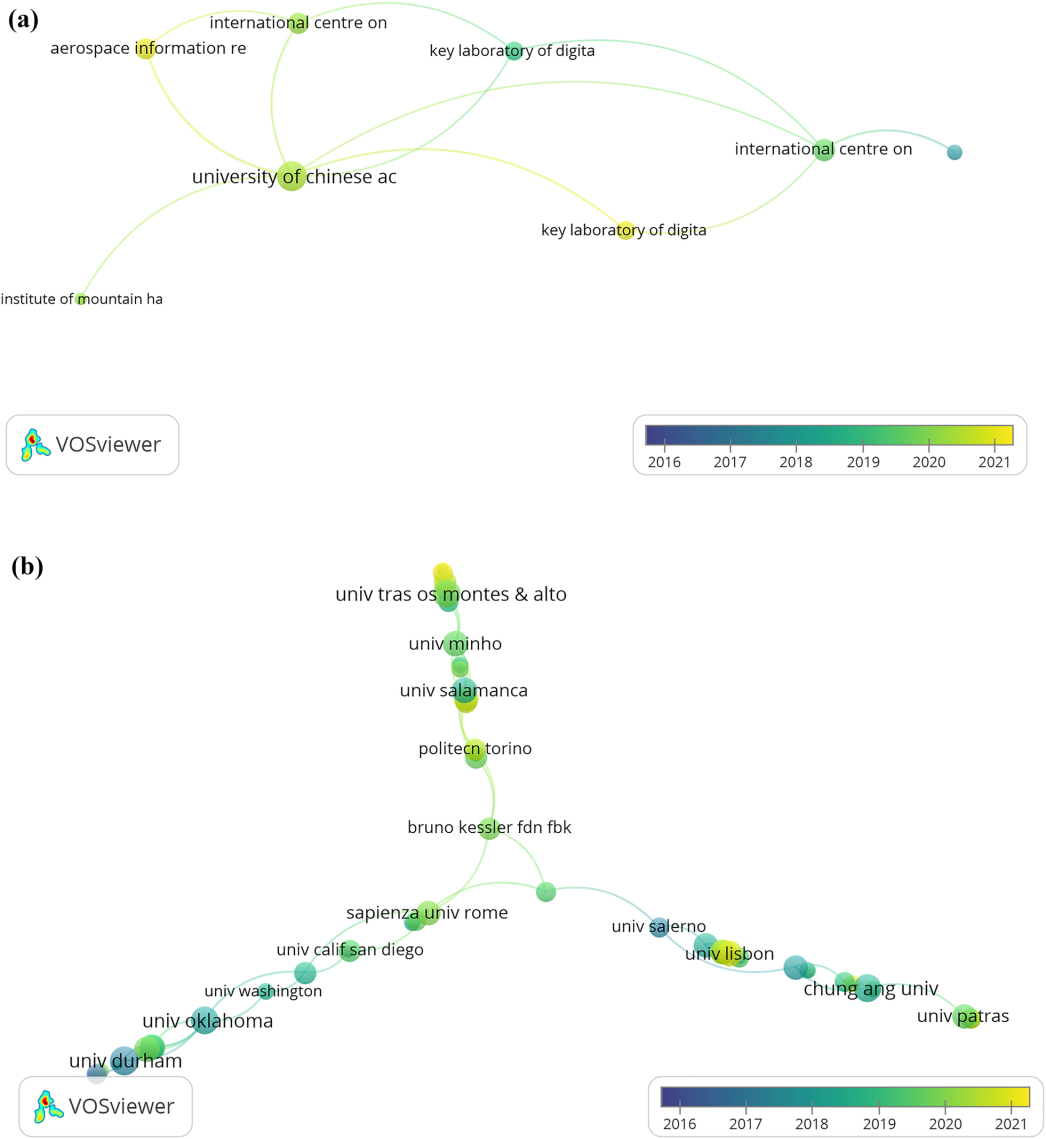
The most collaborative institutions in heritage studies combined with industry 4.0 from 2016 to 2021 in **a** Scopus and **b** WoS in VOSviewer

Meanwhile, from the collaborative point of view, the University of Chinese Academy of Science (UCAS), with six visible nodes (see Fig. 4a) plus 4 hidden (see Table 8 in Appendix B), stands out as the most associative institution according to Scopus. Other bibliometric studies related have obtained a similar outcome [38]. However, the WoS data did not provide visual information in Fig. 4b about the leader institution but showed the limited collaboration of most universities. Also, Appendix B indicates that for WoS, UCAS University occupied the second position, with three links less than the Chinese Academy of Sciences -manager of UCAS-, which reaches 13 connections. Moreover, Fig. 4 points out that new organizations are emerging in the past few years.

### Most participative funding agencies

Scopus and WoS showed differences in their hosted most sponsoring agencies. The top 3 institutions in Scopus doubled the WoS ones. *The Horizon 2020 Framework Programme* is the principal funding agency, which represents the world’s most extensive research and innovation program created by the European Union to support and encourage research in the European Research Area (ERA) [39]. The *European Commission* has played an important role in heritage studies. For instance, it inaugurated the European Year of Cultural Heritage (EYCH) in 2018 to boost engagement with Europe’s past [40]. The *National Natural Science Foundation of China* rounded out the top three (see Table 5), due to its commitment to research in multiple disciplines, which has led to several researchers achieving the Chinese National Natural Science Award [41].

**Table 5:** Top 10 most participative funding agencies in heritage-industry 4.0 research (2016–2021)

Rank	Scopus			WoS		
	Affiliations	Country	No. of paper	Affiliations	Country	No. of paper
1st	Horizon 2020 Framework Programme	Belgium	71	European Commission	Belgium	33
2nd	European Commission	Belgium	61	National Natural Science Foundation of China	China	16
3rd	National Natural Science Foundation of China	China	46	UK Research and Innovation	UK	12
4th	National Science Foundation	USA	34	National Science Foundation	USA	9
5th	European Regional Development Fund	Belgium	28	Spanish Government	Spain	9
6th	Spanish Government	Spain	21	Arts & Humanities Research Council	England	6
7th	Chinese Academy of Sciences	China	19	Ministero dell'Istruzione—Miur	Italy	6
8th	National Aeronautics and Space Administration	USA	16	Ministry of Science and Technology	Taiwan	5
9th	European Resuscitation Council	Belgium	15	Chinese Academy of Sciences	China	4
10th	Ministero dell'Istruzione—Miur	Italy	12	Engineering and Physical Sciences Research Council	UK	4

### Most contributing countries and their collaborations

Both databases were consistent about the leading top countries in heritage studies developed under the outlook of industry 4.0, including at least ten publications per institute in VOSviewer, omitting unconnected nodes. Italy stands as the principal country that has successfully incorporated ideas, technology, and innovation from computers to heritage sciences. This statement is sustained by the number of citations received, which highly overpasses the numbers obtained by China and the United Kingdom (see Table 6). Italy is not only the leader in production but also in the impact of their 4.0 heritage studies. The success of Italy and China must be framed in the implementation of national policies by both governments. Italy launched the ‘Piano Nazionale Industria 4.0’ national plan in 2018, which aimed to support companies investing in new technologies, modernizing machinery, creating new patents, and pushing innovation [42]. China’s actual empowerment was thoroughly planned in the “Made in China 2025” strategy from 2015, which was directed to catch up with industry 4.0 technologies [43].

**Table 6:** Top 10 countries in heritage-industry 4.0 research (2016–2021)

Rank	Scopus			Wos		
	Country	Number of papers	Total citations	Country	Number of papers	Total citations
1st	Italy	406	2059	Italy	306	1074
2nd	China	315	1418	China	182	643
3rd	USA	315	868	USA	159	253
4th	Spain	206	706	Spain	151	297
5th	Uk	194	1444	Uk	131	850
6th	Australia	102	301	Greece	66	120
7th	France	90	192	South Korea	62	339
8th	Germany	88	238	France	57	74
9th	Greece	76	223	Portugal	54	27
10th	Netherlands	66	208	Netherlands	42	61

One of Italy’s recent initiatives is reflected in the resilience of its museums during the COVID-19 pandemic in 2020, where online cultural material and other initiatives migrated to social media -Twitter, Instagram, and Facebook-, showing a sharp rise in their regular online activity [44]. This practice proved the utility of digital technologies to boost the number of visitors, reduce costs, improve the visitor experience, and adapt to competitors. Hence, the COVID-19 pandemic accelerated the adoption of digital technologies. However, more funding is essential for the inclusion of more digital tools [45].

**Fig. 5:**
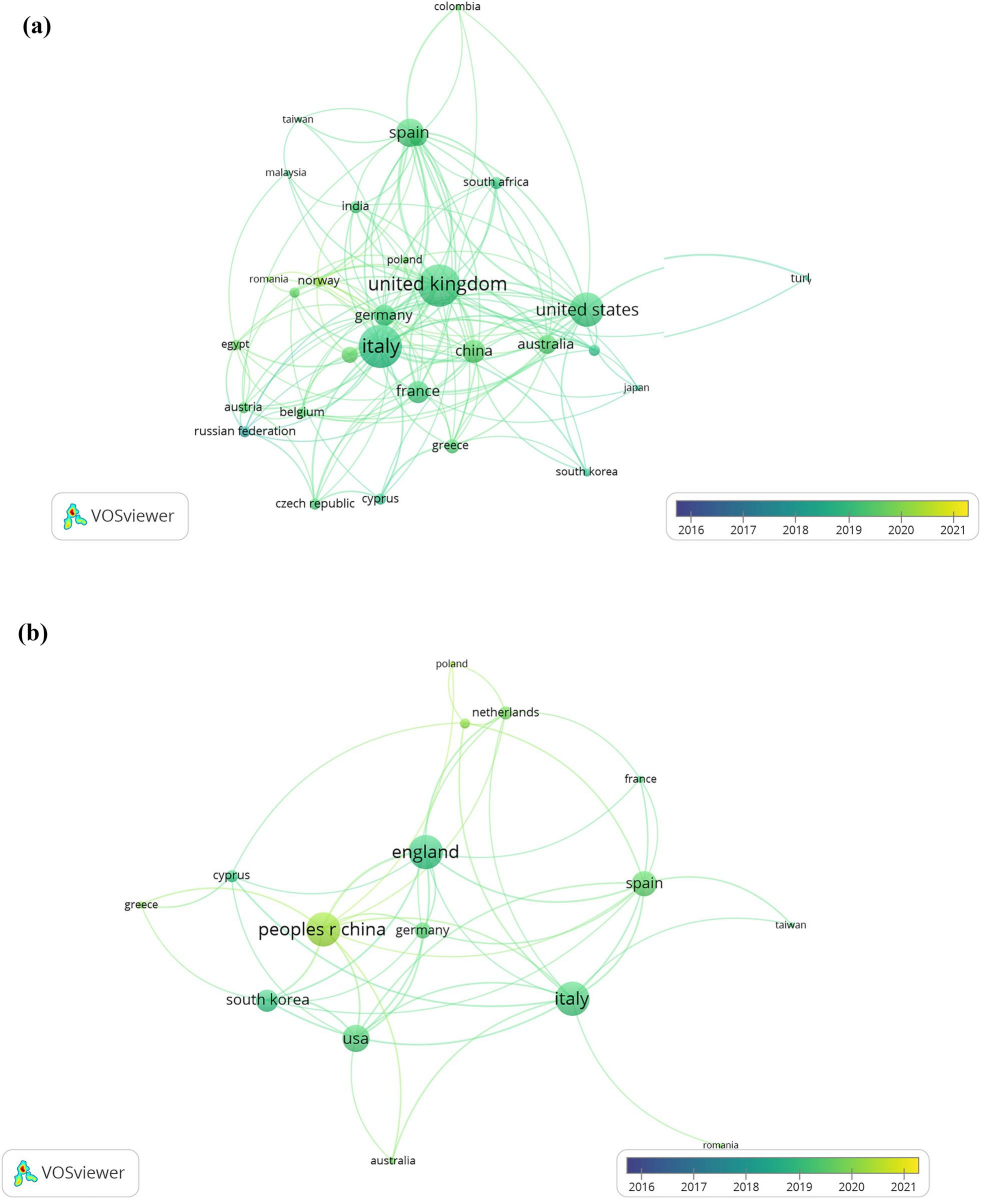
Most collaborative countries in heritage studies combined with industry 4.0 from 2016 to 2021 in **a** Scopus and **b** WoS, considering a minimum of ten documents per country in VOSviewer

Figure 5 shows that the most collaborative countries are Italy and UK whereas USA and China intercalate the third place depending on the database explored (see Table 9 of Appendix C). Otherwise, the same figure shows that China, Spain, Australia, Norway, and Netherlands are countries that have been increasing their collaborations in the past two years.

### Relationship among institutions, countries, and keywords

**Fig. 6:**
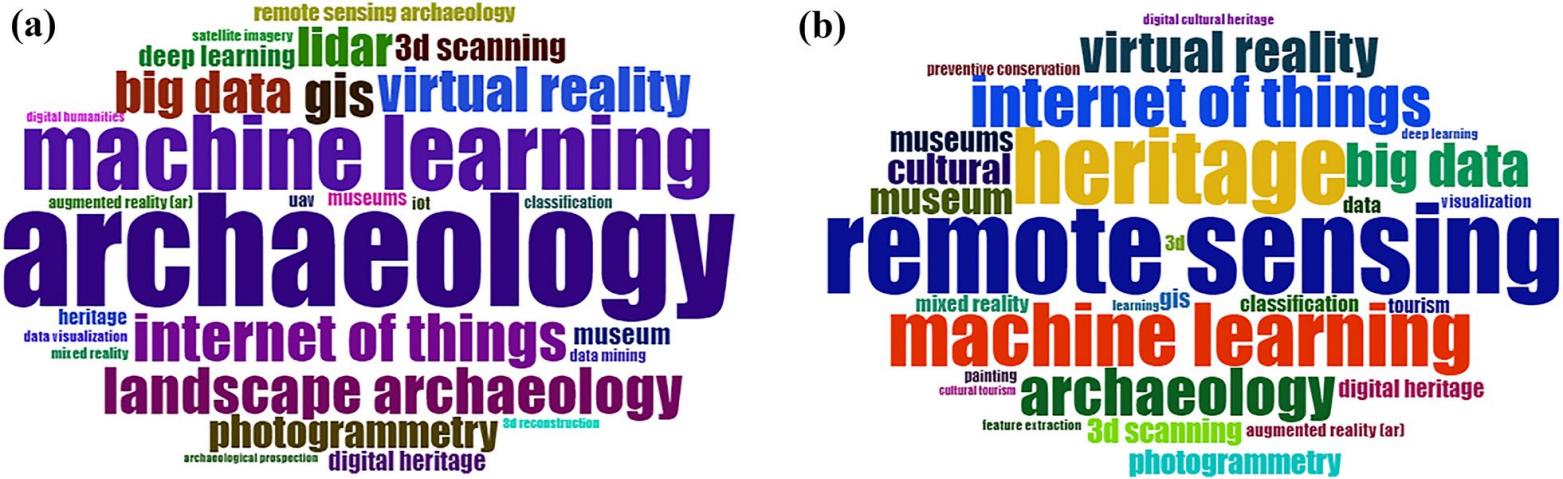
Word clouds of authors’ keywords in heritage studies combined with industry 4.0 from 2016 to 2021 in **a** Scopus and **b** WoS

Keywords are loyal representations of scientific research works in articles, and their frequent implementations may reflect the hotspots of a particular study field. The word-cloud visualization of Scopus and WoS database in *Biblioshiny* (see Fig. 6), allowed us to define the most relevant keyword introduced by authors in heritage studies linked with industry 4.0 technologies. These results highlight that the key terms for heritage science are archaeology, museum, and tourism while those for computer science are remote sensing, augmented reality, and machine learning (see Table 10 in Appendix D).

Remote sensing provides data for mapping the surface of the earth, identification of landslides, and environmental monitoring for cultural heritage decision-making [46]. Augmented reality may combine with virtual reality, since both include electronic devices -tablets, smartphones, laptops, etc. to attract tourists and encourage them to visit museums [47]. Machine learning has been adopted by archaeologists to study geospatial, material cultural, textual, natural, and artistic data. These algorithms are trained to identify and classify archaeological features and objects [48]. Institutions may fluctuate but in general terms, remote sensing and augmented reality are the most prioritized technologies in heritage studies (see Fig. 8 in Appendix D). Furthermore, these technologies can be introduced together for education purposes in several heritage areas [49].

**Fig. 7:**
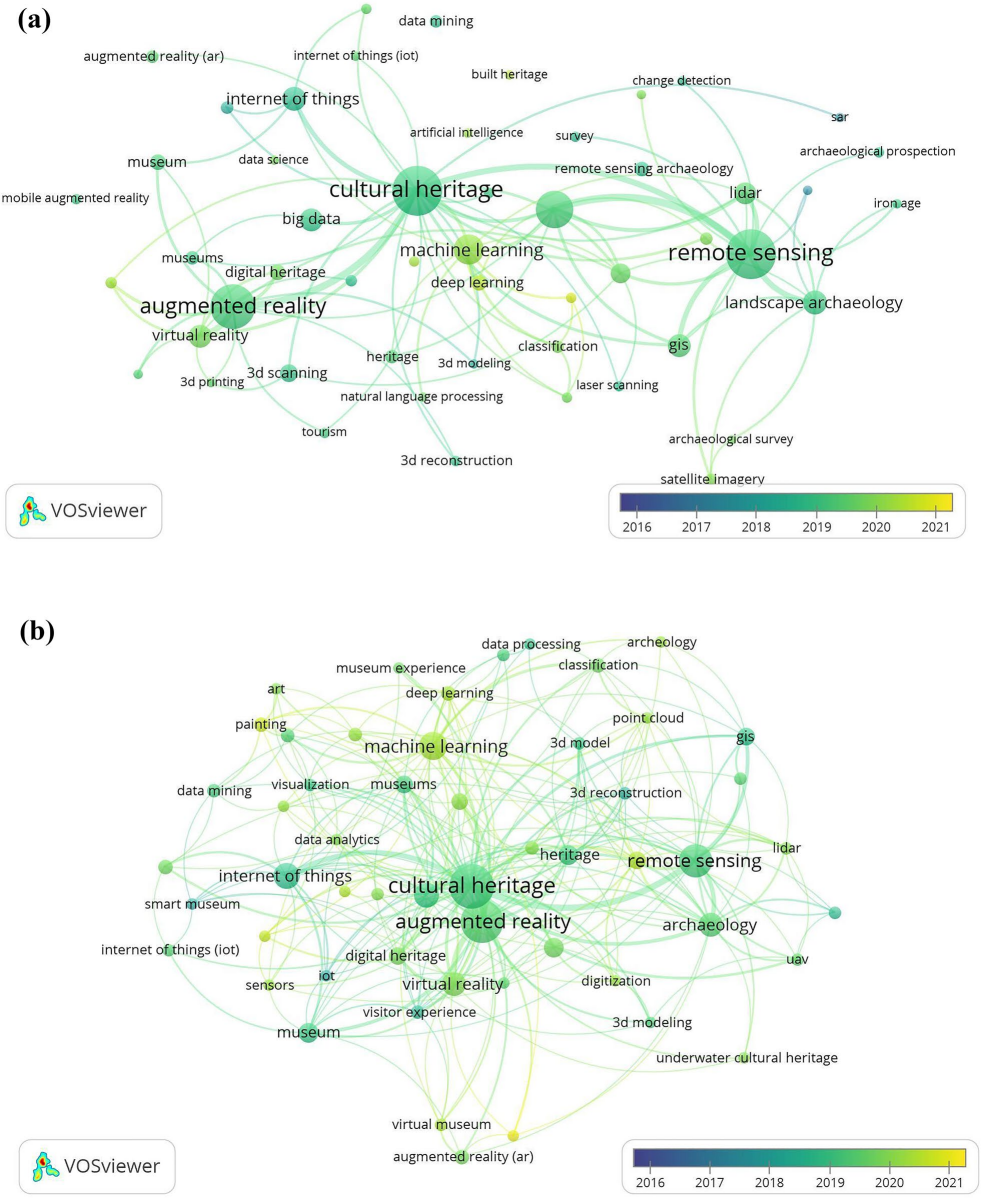
Keywords most used by authors in heritage studies combined with industry 4.0 from 2016 to 2021 in **a** Scopus and **b** WoS, considering a minimum of eight and five occurrences in VOSviewer, respectively

**Fig. 8:**
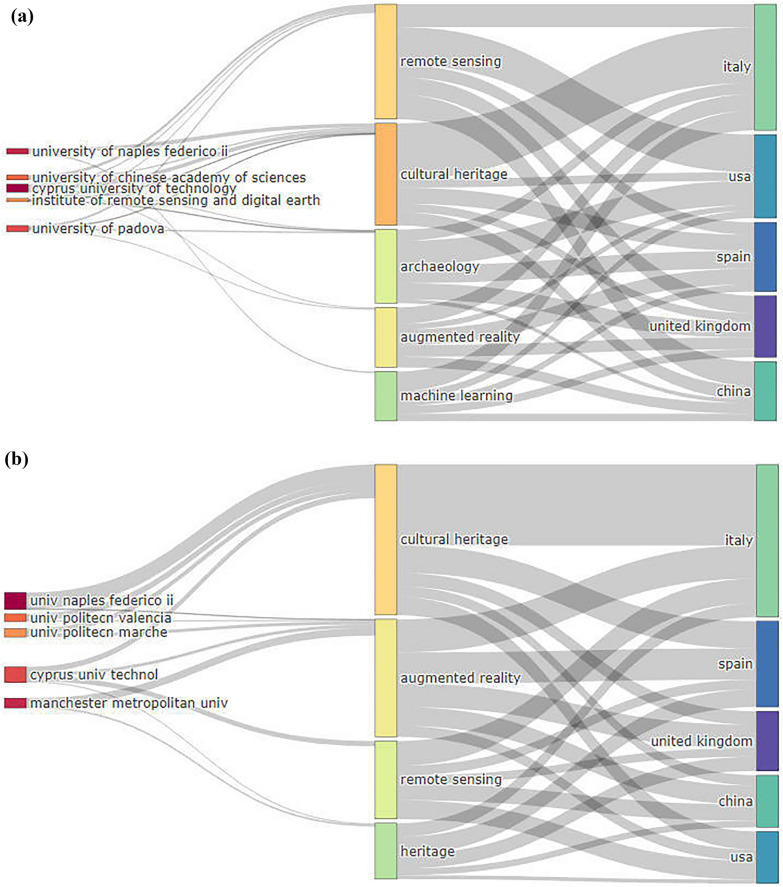
Relationship between institutions, countries, and most used keywords in heritage studies combined with industry 4.0 from 2016 to 2021 in **a** Scopus and **b** WoS

Figure 7 shows that machine learning and deep learning technologies are gaining importance in heritage studies. For instance, social networks are useful for mapping values and attributes transmitted by the public to cultural heritage through the collection of images, texts, geographic locations, and so forth, by means of graph-based machine learning algorithms [50]. Artistic paintings have been analyzed through automatic classification by deep learning, extracting detailed features of oil painting images to provide accurate results [51]. We believe that these applications belong to new pathways of heritage studies addressed to industry 4.0, and serve as indicators for future routes to be explored by scientists.

## Conclusions

This bibliographic review demonstrates the recent growing interest shown by institutions, journals, researchers, countries, and funding agencies in the study of cultural heritage associated with the use of technological tools provided by industry 4.0. The main conclusions delivered by responding to each one of the settled research questions are the following:

- The production of original papers in the explored fields is currently under linear growth.
- A minimum of 4 published papers, with more than one hundred citations, is required to become one of the most cited authors on the tracked type of research.
- The most cited articles on these issues deal with augmented reality and IoT applications in cultural heritage.
- The main magazines that disseminate industry 4.0 initiatives linked to cultural heritage have a JCR greater than 2, with remote sensing being the preferred repository.
- The most productive institutions delivered at least 10 documents to be part of the top ten.
- Funding agencies pursuing the top ten of given awards needed to finance a minimum of 4 papers.
- Italy and China are the most concerned countries regarding the fourth industrial revolution applied to material science, whose success stems from the incorporation of specific public policies.
- Augmented reality and remote sensing represent the most attractive technologies to perform new studies in heritage science.

In general terms, this bibliometric analysis offers an updated standpoint on heritage sciences for developing subsequent research and generating more consciousness about the impact of introducing new technologies in the promotion, discovery, management, and conservation of world cultural and historical heritage. Although augmented reality and remote sensing are the research hotspots technologies in heritage studies, and they have become usual in archeology and museums to trigger education and tourism, exist future possible pathways such as machine learning and deep learning that are as well used in heritage areas like artistic paintings. Finally, we suggest that future research will validate the quantitative trends found in each of the addressed questions presented in the introduction section.

## Data Availability

The databases used can be freely downloaded from the Web of Science and Scopus websites following the procedure explained in the methodology section.

## Appendix A

See Table 7.

**Table 7:** Top 5 collaborative authors in heritage-industry 4.0 research (2016–2021)

Rank	Scopus			WoS		
	Author	No. of paper	T. link strength	Author	No. of paper	T. link strength
1st	Lasaponara R	16	67	Piccialli F	9	16
2nd	Piccialli F	15	32	Dieck MCI	8	10
3rd	Chen F	13	63	Chianese A	7	11
4th	Masini N	11	52	Ceika J	4	12
5th	Wang X	8	35	Liarokapis F	4	12

## Appendix B

See Table 8.

**Table 8:** Top 5 collaborative institutions in heritage-industry 4.0 research (2016–2021)

Rank	Scopus			WoS		
	Institution	No. of paper	T. link strength	Institution	Art	No. of paper
1st	Univ Chinese Acad	12	10	Chinese Acad Sci	10	13
2nd	Int Cen on Spa Tech	4	5	Univ Chinese Acad	7	10
3rd	Aero Inf Res Inst	4	4	Cyprus Univ Tech	9	9
4th	Cent for Rock Art Res	2	4	Univ Durham	4	8
5th	Col of Hum, Art & Soc	2	4	Univ Babeș-Bolyai	4	7

## Appendix C

See Table 9.

**Table 9:** Top 5 collaborative countries in heritage-industry 4.0 research (2016–2021)

Rank	Scopus		WoS	
	Country	Total, link strength	Country	Total, link strength
1st	Italy	101	UK	23
2nd	UK	96	Italy	23
3rd	USA	71	China	23
4th	Spain	55	USA	18
5th	China	42	Spain	17

## Appendix D

See Table 10, Fig. 8.

**Table 10:** Top 5 most used keywords in heritage-industry 4.0 research (2016–2021)

Rank	Scopus		WoS	
	Terms	Frequency	Terms	Frequency
1st	Cultural heritage	195	Cultural heritage	122
2nd	Remote sensing	193	Augmented reality	119
3rd	Augmented reality	154	Remote sensing	60
4th	Archaeology	109	Heritage	50
5th	Machine learning	73	Machine learning	42
